# Supplementary material for: ESCRTs function directly on the lysosome membrane to downregulate ubiquitinated lysosomal membrane proteins
Source: eLife. 2017 Jun 29;6:e26403. doi: 10.7554/eLife.26403 (PMC5507667; doi:10.7554/eLife.26403)
Supplement: Supplementary file 2. — DOI: http://dx.doi.org/10.7554/eLife.26403.020 [file elife-26403-supp2.docx]

Supplemental file 2: The mutants defective for Ypq1 sorting found by ‘spontaneous mutagenesis’ and ‘fluorescence screen’.

| **Category** | **Mutant** | **Spontaneous mutagenesis** | **Fluorescence screen** |
| --- | --- | --- | --- |
| E3 ligase complex | *ssh4* | x | x |
|  | *rsp5* | x |  |
| ESCRT-0 | *vps27* | x | x |
|  | *hse1* | x | x |
| ESCRT-I | *vps37* | x | x |
|  | *vps23* | x | x |
|  | *vps28* | x | x |
| ESCRT-II | *vps25* | x | x |
|  | *vps36* | x | x |
|  | *vps22* | x | x |
| ESCRT-III | *bro1* | x | x |
|  | *vps20* | x | x |
|  | *snf7* | x | x |
|  | *vps24* | x | x |
|  | *vps2* | x | x |
| ESCRT accessory | *did2* | x | x |
|  | *vps60* | x | x |
| ESCRT disassembly | *Vps4* | x | x |
| CORVET/ HOPS | *vps11* |  | x |
|  | *vps16* |  | x |
|  | *vps18* |  | x |
|  | *vps33* | x | x |
|  | *vps39* |  | x |
|  | *vps41* |  | x |
|  | *vps3* |  | x |
|  | *vps8* |  | x |
| SNAREs | *pep12* |  | x |
|  | *vam3* |  | x |
|  | *vam7* |  | x |
| PdtIns(3)P Kinase | *vps15* |  | x |
|  | *vps34* |  | x |
