# Supplementary material for: ESCRTs function directly on the lysosome membrane to downregulate ubiquitinated lysosomal membrane proteins
Source: eLife. 2017 Jun 29;6:e26403. doi: 10.7554/eLife.26403 (PMC5507667; doi:10.7554/eLife.26403)
Supplement: Supplementary file 3. — DOI: http://dx.doi.org/10.7554/eLife.26403.021 [file elife-26403-supp3.docx]

Supplemental file 3: Supplementary Experimental Materials

A. Yeast strains used in this study

| **Strains** | **Genotype** | **Use** | **Source** |
| --- | --- | --- | --- |
| SEY6210 | *MATα, leu2-3,112 ura3-52 his3-*Δ*200 trp1-Δ901 lys2-801 suc2-*Δ*9* | Throughout | (Robinson et al., 1988) |
| SEY6210.1 | *MATa, leu2-3,112 ura3-52 his3-*Δ*200 trp1-Δ901 lys2-801 suc2-*Δ*9* | Throughout | (Robinson et al., 1988) |
| SEY6211 | *MATα, leu2-3,112 ura3-52 his3-*Δ*200 trp1-Δ901 ade2-101 suc2-*Δ*9* | Throughout | (Robinson et al., 1988) |
| BY4741 knock-out collection | *MATa his3Δ1 leu2*Δ*0 met15*Δ*0 ura3*Δ*0, each gene was knocked out by KAN.* | Figure S1 | GE Dharmacon |
|  |  | Figure 1 |  |
| BHY154 | *SEY6210, Vph1-mCherry::TRP* | Figure 1A | (Han and Emr, 2011) |
| LZY376 | *SEY6210, Vph1-mCherry::TRP, ssh4*Δ*::HPH* | Figure 1B | This study |
| LZY137 | *SEY6210, Ypq1-SBP-GFP-HIS3::LEU* | Figure 1D | This study |
| LZY138 | *SEY6211, Ypq1-SBP-GFP-HIS3::LEU* | Figure 1D | This study |
|  |  | Figure2 |  |
| YMB482 | *SEY6210, vps27*Δ*::HIS3*, *Vph1-mCherry::TRP1* | Figure 2A | This study |
| JJY162 | *SEY6210, Ypq1-GFP::TRP1, doa4*Δ*:KAN, pRS426-Myc-Ub* | Figure 2B | This study |
| JJY165 | *vps27*Δ*::HIS3, Ypq1-GFP::TRP1, doa4*Δ*::KAN, pRS426-Myc-Ub* | Figure 2B | This study |
| JJY111 | *SEY6210.1, tor1-1, fpr1*Δ*::NAT, pRS305-pGPD-FRB-3xUb::LEU, Ypq1-GFP-2xFKBP::TRP, vph1-mCherry::TRP* | Figure 2D, 2E | This study |
| JJY106 | *SEY6210.1, tor1-1, fpr1*Δ*::NAT, pRS305-pGPD-FRB-3xUb::LEU, Ypq1-GFP-2xFKBP::TRP* | Figure 2F | This study |
| LZY496 | *SEY6210.1, tor1-1, fpr1*Δ*::NAT, pRS305-pGPD-FRB-3xUb::LEU, Ypq1-GFP-2xFKBP::TRP, ssh4*Δ*::KAN* | Figure 2F | This study |
|  |  | Figure3 |  |
| JJY149 | *SEY6210.1, tor1-1, fpr1*Δ*::NAT, pRS305-pGPD-FRB-3xUb::LEU, Ypq1-GFP-2xFKBP::TRP, vps18Δ::Trp1, vps18ts::URA, vph1-mCherry::KAN* | Figure 3A, 3B | This study, |
| LZY445 | *SEY6210.1, tor1-1, fpr1*Δ*::NAT, pRS305-pGPD-FRB-3xUb::LEU, Ypq1-GFP-2xFKBP::TRP, vam7Δ::KAN, vam7ts::URA, vph1-mCherry::HPH* | Figure 3C, 3D | This study |
| JJY135 | *SEY6210.1, tor1-1, fpr1*Δ*::NAT, pRS305-pGPD-FRB-3xUb::LEU, Ypq1-GFP-2xFKBP::TRP, sec18ts, vph1-mCherry::TRP1* | Figure 3E, 3F | This study |
|  |  | Figure4 |  |
| LZY394 | *SEY6210.1, tor1-1, fpr1*Δ*::NAT, pRS305-pGPD-FRB-3xUb::LEU,Ypq1-GFP-2xFKBP::TRP, vps4*Δ*::HPH, pRS306-vps4ts::URA, Vph1-mCherry::KAN* | Figure 4A,4B | This study |
| YMB444 | *SEY6210, Vph1-mCherry::TRP, vps34*Δ*::HIS* | Figure 4C | This study |
|  |  | Figure5 |  |
| LZY263 | *SEY6210.1, tor1-1, fpr1*Δ*::NAT, pRS305-pGPD-FRB-3xUb::LEU, Ypq1-GFP-2xFKBP::TRP, vps27*Δ*::HPH* | Figure 5D | This study |
|  |  | Figure6 |  |
| LZY700 | *SEY6210.1, tor1-1, fpr1*Δ*::NAT, pRS305-pGPD-FRB-3xUb::LEU, Ypq1-GFP-2xFKBP::TRP, Hse1-mCherry::HPH* | Figure 6A | This study |
| LZY397 | *SEY6210.1, tor1-1, fpr1*Δ*::NAT, pRS305-pGPD-FRB-3xUb::LEU, Ypq1-GFP-2xFKBP::TRP, Vps23-mCherry::HPH* | Figure 6B | This study |
|  |  | Figure7 |  |
| LZY448 | *SEY6210.1, tor1-1, fpr1*Δ*::NAT, pRS305-pGPD-FRB-3xUb::LEU, Ypq1-GFP-2xFKBP::TRP, pep12*Δ*::HIS, pep4*Δ*::HPH, atg9*Δ*::KAN, vma4*Δ*::KAN,* | Figure 7A, 7E, 7F | This study |
| LZY463 | *SEY6210.1, tor1-1, fpr1*Δ*::NAT, pRS305-pGPD-FRB-3xUb::LEU, Ypq1-GFP-2xFKBP::TRP, pep12Δ::HIS, pep4*Δ*::HPH, atg9*Δ*::KAN, vma4*Δ*::KAN, pRS306-Hse1DUB::URA* | Figure 7B, 7C, 7E, 7F | This study |
| LZY471 | *SEY6210.1, tor1-1, fpr1*Δ*::NAT, pRS305-pGPD-FRB-3xUb::LEU, Ypq1-GFP-2xFKBP::TRP, pep4Δ::HPH, atg9*Δ*::KAN, vma4*Δ*::KAN* | Figure 7D, 7F | This study |
|  |  | Figure2-figure supplemental 1 |  |
| TDY27 | *SEY6210, vam3ts* | Figure2-f.s.1A | (Abeliovich et al., 1999) |
| SRY18T-1 | *SEY6210, vps18ts* | Figure2-f.s.1A | (Rieder and Emr, 1997) |
| JJY195 | *SEY6210, Ypq1-GFP::Kan, vps18ts, doa4*Δ*::His3, pRS426-Myc-Ub* | Figure2-f.s.1B | This study |
| JJY196 | *SEY6210, Ypq1-GFP::TRP1*, *vam3*Δ*::His3, vam3ts::LEU2*, *doa4*Δ::*KAN, pRS426-Myc-Ub* | Figure2-f.s.1B | This study |
|  |  | Figure2-figure supplemental 2 |  |
| LZY701 | *SEY6210.1,MATa, tor1-1, fpr1Δ::NAT, Ypq1-GFP-2xFKBP::TRP, Vph1-mCherry::TRP* | Figure2-f.s.2 | This study |
|  |  | Figure2-figure supplemental 4 |  |
| LZY562 | *SEY6210, tor1-1, fpr1*Δ*::NAT, pRS305-pGPD-FRB-3xUb::LEU, Can1-GFP-2xFKBP::TRP, Vph1-mCherry::KAN* | Figure2-f.s.4A,B | This study |
| LZY561 | *SEY6210, tor1-1, fpr1*Δ*::NAT, pRS305-pGPD-FRB-3xUb::LEU, Kex2-GFP-2xFKBP::TRP, Vph1-mCherry::KAN* | Figure2-f.s.4C,D | This study |
| LZY560 | *SEY6210, tor1-1, fpr1*Δ*::NAT, pRS305-pGPD-FRB-3xUb::LEU, Vph1-GFP-2xFKBP::TRP, pLZ476* | Figure2-f.s.4E,F | This study |
| LZY564 | *SEY6210, tor1-1, fpr1*Δ*::NAT, pRS305-pGPD-FRB-3xUb::LEU, Nhx1-GFP-2xFKBP::TRP, Vph1-mCherry::KAN* | Figure2-f.s.4G,H | This study |
|  |  | Figure3-figure supplemental 1 |  |
| LZY630 | *SEY6210, Vph1-mCherry::TRP, pRS305-Mup1-GFP::LEU* | Figure3- f.s. 1A,B,C | This study |
| JJY171 | *SEY6210, Vph1-mCherry::KAN, pRS305-Mup1-GFP::LEU, , vps18*Δ*::TRP1, vps18ts::URA* | Figure3- f.s. 1A,B | This Study, |
| JJY170 | *SEY6210, Vph1-mCherry::TRP, pRS305-Mup1-GFP::LEU, sec18ts* | Figure3- f.s. 1A,C | This study |
| TKY025 | *SEY6210, Mup1-GFP::KAN, Vph1-mCherry::TRP* | Figure3- f.s. 1D | This study |
| LZY570 | *SEY6210, Mup1-GFP::KAN, Vph1-mCherry::TRP, vam7*Δ*::KAN, vam7ts::URA,* | Figure3- f.s. 1A,D | This study |
|  |  | Figure6-figure supplemental 1 |  |
| LZY703 | *SEY6210.1, tor1-1, fpr1*Δ*::NAT, pRS305-pGPD-FRB-3xUb::LEU, Ypq1-GFP-2xFKBP::TRP, Vps36-mCherry::HPH* | Figure6- f.s. 1 | This study |
|  |  | Figure6-figure supplemental 3 |  |
| LZY395 | *SEY6210.1, tor1-1, fpr1*Δ*::NAT, pRS305-pGPD-FRB-3xUb::LEU, Ypq1-GFP-2xFKBP::TRP, Vps23-mCherry::KAN, vps4*Δ*::HPH, pRS306-vps4ts::URA, pLZ698* | Figure6- f.s. 3A,B | This study |

B. Plasmids used in this study

| **Name** | **Description** | **Use** | **Source** |
| --- | --- | --- | --- |
| MLP379 | pFA6a-GFP-2xFKBP::TRP | Throughout | This study |
| MLP479 | pRS305-pGPD-FRB-3xUb | Throughout | This study |
|  |  | Figure 1 |  |
| MLP350 | pRS416-Ypq1-SBP-GFP | Figure 1A,B | This study |
| pLZ64 | pRS305-Ypq1-SBP-GFP-His3 | Figure 1D | This study |
|  |  | Figure 2 |  |
| MLP280 | pRS426-Myc-Ub | Figure 2B | This study |
|  |  | Figure 3 |  |
| pLZ730 | pRS306-vam7ts | Figure 3C,D | This study and (Sato et al., 1998) |
|  |  | Figure 4 |  |
| pLZ336 | pRS306-vps4ts | Figure 4A,B | This study and (Babst et al., 1997) |
| pLZ562 | pRS416-pADH1-GFP-2xFYVE (from Vps27, 165-231) | Figure 4C | This study |
|  |  | Figure 7 |  |
| pPL4586 | pRS306-Cup1-Hse1UL36-3HA | Figure 7B,C,D,E | (MacDonald et al., 2012) |
|  |  | Figure 1-figure supplemental 1 |  |
| MLP359 | pRS416-Ypq1-sbp-pHluorin | Figure 1-f.s. 1 | This study |
|  |  | Figure 2-figure supplemental 2 |  |
| pLZ576 | pRS416-Ypq1-mCherry | Figure 2-f.s. 3A,B,C | This study |
| pLZ476 | pRS416-pPRC1-mCherry-ALP | Figure 2-f.s. 4E | This study |
|  |  | Figure 6-figure supplemental 3 |  |
| pLZ698 | pRS413-pCUP1-Hse1(Δ288-375)-UL36 | Figure 6-f.s. 3A,B | This study |

Supplemental References:

Abeliovich, H., T. Darsow, and S.D. Emr. 1999. Cytoplasm to vacuole trafficking of aminopeptidase I requires a t-SNARE-Sec1p complex composed of Tlg2p and Vps45p. EMBO J. 18:6005-6016.

Babst, M., T.K. Sato, L.M. Banta, and S.D. Emr. 1997. Endosomal transport function in yeast requires a novel AAA-type ATPase, Vps4p. *EMBO J*. 16:1820-1831.

Han, B.K., and S.D. Emr. 2011. Phosphoinositide [PI(3,5)P2] lipid-dependent regulation of the general transcriptional regulator Tup1. *Genes Dev*. 25:984-995.

MacDonald, C., N.J. Buchkovich, D.K. Stringer, S.D. Emr, and R.C. Piper. 2012. Cargo ubiquitination is essential for multivesicular body intralumenal vesicle formation. *EMBO Rep*. 13:331-338.

Rieder, S.E., and S.D. Emr. 1997. A novel RING finger protein complex essential for a late step in protein transport to the yeast vacuole. Mol Biol Cell. 8:2307-2327.

Robinson, J.S., T.R. Graham, and S.D. Emr. 1991. A putative zinc finger protein, Saccharomyces cerevisiae Vps18p, affects late Golgi functions required for vacuolar protein sorting and efficient alpha-factor prohormone maturation. *Mol Cell Biol*. 11:5813-5824.

Robinson, J.S., D.J. Klionsky, L.M. Banta, and S.D. Emr. 1988. Protein sorting in Saccharomyces cerevisiae: isolation of mutants defective in the delivery and processing of multiple vacuolar hydrolases. *Mol Cell Biol*. 8:4936-4948.

Sato, T.K., T. Darsow, and S.D. Emr. 1998. Vam7p, a SNAP-25-like molecule, and Vam3p, a syntaxin homolog, function together in yeast vacuolar protein trafficking. *Mol Cell Biol*. 18:5308-5319.
